# Analyzing nurses’ decisions to leave their profession—a duration analysis

**DOI:** 10.1007/s10198-023-01600-y

**Published:** 2023-06-07

**Authors:** Martin Kroczek

**Affiliations:** Institute for Applied Economic Research (IAW), Tübingen, Germany

**Keywords:** Nurses’ labor supply, Duration analysis, Occupation duration, J63, J62, I18

## Abstract

Many countries suffer from skilled labor shortages in nursing. One way to increase the nurse labor supply is to raise their retention rates. Yet, though several studies exist on factors associated with the nurse labor supply at different levels, literature on factors associated with nurses’ decisions to leave their occupation is relatively scarce. Based on German administrative data, I analyze the determinants of nurses’ decisions to leave their profession. My results suggest that younger nurses, nurses in the social sector, and nurses working with smaller employers leave their occupation more often than their counterparts, irrespective of their specific nursing occupations and care settings. Nurses leave more often where more alternative occupational options are available. Nurses who have been unemployed and nurses who have been employed in a different field have a higher probability of leaving the occupation, whereas nurses who just finished vocational training only have a moderate propensity to leave. Female nurses leave less often if employed part time. Female nurses in part time leave even more seldom if they have children. A change in the hospital reimbursement system and introducing a nursing minimum wage during the first decade of the century did not change nurses’ occupation durations.

## Introduction

Many countries have been suffering from skilled labor shortages in nursing occupations for a couple of years now [27]. In Germany, employers were not able to fill open health nurse positions within 168 days and geriatric nurse positions within 210 days on average in 2020 [16]. Several countries undertook measures to increase the nurse labor supply, such as a push for increased immigration of nurses, changes in nursing training or implementing new occupational profiles [15, 51]. Germany, for example, introduced a nationwide action plan, the Concerted Action on Nursing [17]. The shortage in nursing personnel has become a severe problem during the COVID-19 pandemic [8, 24, 50]. One strategy to increase the supply of nurses is to raise their retention rates. Yet, though several studies exist on factors associated with the nursing labor supply on different levels, literature on factors associated with nurses’ decisions to leave their occupation—and therefore, guidance for corresponding policies—is relatively scarce [27].

This paper contributes to the literature on factors associated with nurses’ decisions to leave their occupation. Though there exist previous analyses on factors influencing nurses’ labor supply in general as well as their intention and actual decision to leave nursing jobs or the health care system, evidence on factors driving nurses’ decision to leave the occupation entirely is scarce, focuses on specific leaving events, or relies on less rich and less precise data or relatively short time horizons. In this study, I use highly reliable and precise German administrative data, which contain labor market biographies of a 2-percent sample of all individuals in jobs subject to social security payments, augmented with data on regional characteristics. In my econometric analysis, I apply survival analysis methods to daily spell data, allowing me to estimate the impacts of individual, firm, and regional characteristics on nurses’ exit behavior while accounting for duration dependence. I pay special attention to factors that are subject to adjustments by employers and political players, such as wages. Other factors of special interest are those that help identify situations and groups associated with particularly high risks of exiting the occupation, such as characteristics of the regional labor market and nurses’ prior labor market experience. Unlike previous studies, I can account for specific events in individuals’ labor market biographies in my analysis, not just general work experience, and I find differences in the propensity to leave between individuals who enter immediately after vocational training, re-enter after the birth of a child, or have had periods of work or unemployment of different lengths in the recent past. I aim to provide evidence on where action is needed to keep nurses in their occupation. From a public policy standpoint, it may be problematic if nurses leave their occupation in general. It may, however, be more worrying if they leave into unemployment rather than to another work in the health and social sector. Therefore, I do not only apply single risk analysis but also competing risks analysis to study how different leaving events are associated with different personal, regional, and job characteristics. The results point to remarkable differences in the effects of individual, job, and regional characteristics, most prominently previous labor market experience, on the different leaving propensities. Last, I briefly look at two institutional changes associated with nursing that occurred during the first decade of the twenty-first century: the introduction of the system of diagnosis-related groups (DRGs) and the introduction of a minimum wage for nurses in Germany. Neither change appears to have significantly influenced nurses’ occupational durations.

The rest of the paper is structured as follows: In “Literature”, I summarize the relevant literature on nurses’ decisions to supply labor, focusing on studies that analyze factors associated with the *intention* and the *actual decision* to leave a nursing job, the health sector or even the occupation. In “Data”, I present the data. In “Econometric Methods”, I present the econometric methods used. “Results” contains the results of univariate and multivariate survival analyses. “Conclusion” concludes.

## Literature

The shortage of nurses in various countries has put the nurses’ labor supply on the international research agenda. Three strands of literature are particularly relevant for my analyses. One strand focuses on actual, former, and potential nurse *labor supply in general.* Two other strands focus on the *intention* and, alternatively, the *actual decision* of nurses to leave nursing or the health care system.^1^

The literature on the nurse *labor supply in general* focuses on wage effects on supply, with ambiguous findings. Shields [58] and Antonazzo et al. [3] provide overviews of earlier research in economics on nurse labor supply. Shields [58] concludes that labor supply is rather unresponsive to wage changes, and deduces that non-pecuniary job aspects are of great importance. Di Tommaso et al. [25] and Andreassen et al. [2] reach similar conclusions in recent Norwegian studies, too. Furthermore, Andreassen et al. [2] find differences in wage elasticity across job types and greater labor market mobility for younger nurses (and older nurses to some extent). Moreover, Di Tommaso et al. [25] estimate different labor supply elasticities for different parts of the health care system, opening possibilities to shift labor within the system via wage changes (e.g. between different jobs and between daytime work and shift work). Antonazzo et al. [3], on the contrary, conclude that the effect of one’s *own* wages on labor supply is rather ambiguous, whereas a spouse’s or household income and the presence of very young children negatively affect labor supply. Hanel et al. [39] differentiate between shift types and occupations in their estimation model and account for labor supply decisions on intensive and extensive margins. They find significantly greater wage elasticity of labor supply for nursing degree holders in their Australian survey data than earlier studies that did not make that distinction [39].

The *intention to leave one’s employer* is associated with personal and job characteristics. Kankaanranta and Rissanen [49] find associations among one’s own wage and the share of income from shift work, the possibility for specialization, monotony of work, and excessive duties for a sample of Finnish registered nurses. Beecroft et al. [7] analyze data on newly employed pediatric nurses in the US. They find that older respondents who did not get their ward choice, as well as respondents who worked in institutions with lower environmental and organizational characteristics scores (such as control over practice, opportunities for advancement, workplace ties, and relationships) and those who sought more social support were more likely to exhibit turnover intent.

The intention to leave an employer is also associated with job satisfaction. Kankaanranta and Rissanen [49] establish this connection indirectly via structural modeling techniques. Shields and Ward [59] focus on nurses’ intentions to leave the British National Health Service (NHS) and explicitly take the association between job satisfaction and intent to leave into account. Analyzing data from a 1994 national survey of British NHS nurses, they find overall job satisfaction and satisfaction with different job aspects to be important determinants of the intention to quit working in the NHS. Satisfaction with promotion and training opportunities are of particular importance, whereas satisfaction with workload and pay are less important. They further find that younger nurses, better educated nurses, highly specialized nurses, and members of ethnic minorities want to quit the NHS more frequently.

Other than the formerly named studies, Parry [55] and Simon et al. [60] analyze the intention to leave the *profession* and the intention to leave one’s *employer*, with one result being that factors partially differ between those two outcomes. For the German sample of the Nurses’ Early Exit Study, Simon et al. [60] find that age, burnout, professional commitment and job satisfaction are associated with both the intention to leave one’s profession and employer. The intention to leave one’s profession is further found to be associated with marital status, weekly working hours, and work–family conflict. Intention to leave an organization is further associated with organizational and local characteristics.^2^ For newly graduated nurses in Australia, Parry [55] finds a statistically significant relation between professional and organizational commitment and intent to leave the profession. Furthermore, she finds that the intention to change employers is statistically significantly related to job satisfaction, organizational commitment, and the intention to leave the occupation.

The *intention* to leave one’s employer or profession may differ from the actual leaving decision. Using Kaplan–Meier estimation, Beecroft et al. [7] find that the overwhelming majority of those having voiced turnover intention were still with their original employer after 24 months. Only a few studies focus on nurses’ *actual leaving decision*s, some of which analyze the decision to leave the public health care system. Holmås [44] analyzes Norwegian public sector nurses’ employment durations by employing a discrete time-proportional hazard framework. Controlling for the fact that wages may partly be a compensation for unfavorable working hours (shifts), he finds that wages and working conditions affect nurses’ propensities to leave public sector nursing. Employing data from the UK’s Quarterly Labour Force Survey, Frijters et al. [33] analyze factors associated with nurses’ NHS employment durations. Applying single-risk models and a competing-risks model, Frijters et al. [33] find a significant but small wage effect on retention rates and conclude that nurses’ working conditions (which are assumed to be less pleasant in the NHS than with other employers) are of greater importance regarding the decision to leave the NHS. Furthermore, they find younger nurses, nurses with shorter duration of employment in the NHS, nurses in managerial positions, and nurses employed in smaller establishments show a higher propensity to leave the NHS.

Some studies analyze the decision to leave the profession. A part of this literature focuses on specific leaving events—such as early retirement [32, 54], leaving within the first years of practice [36], or time to exit after training [22]—or concern specific differentiations in leaving behavior [23]. Several studies analyze factors associated with nurses’ actual exit from the profession on a broader basis. Doiron and Jones [26] employ administrative data on registered nurses in New South Wales, and analyze which of the nurses registered in 1996 were still registered one year later. Doiron and Jones [26] find that nurses working in larger hospitals, hospitals with larger nursing staff, and hospitals with higher expenditures are less likely to leave. Higher age, more experience, and more hours of work are associated with lower leaving probability, whereas being male is associated with a higher leaving probability [26]. Employing data from a European one-year survey, Rongen et al. [57] find that reduced work ability, being female, and young age are associated with higher probability of leaving the profession. Nooney et al. [53] apply retrospective survival analysis, where data on professional history were collected as part of a survey from a cross-section of U.S. nurses. They find that nurses change careers more often if they have higher income, children at home, a higher nursing degree, or are female. Other than that, studies on nurses actually leaving their profession are scarce.^3^

This study adds to the literature on job and occupation duration in occupations strongly affected by skill shortages. I further contribute to the literature on nursing supply. My results are of interest, as multivariate analyses on factors associated with nurses exiting their occupation are especially rare. Those that exist are based on less rich or less precise data. They are of significance to policymakers and employers, as they yield important clues on possibilities to keep nurses in their occupation and provide relevant information for recruiting and rehiring processes. Finally, all existing studies either focus solely on nurses in health care or do not differentiate between nurses in health and nurses in geriatric care. To the best of my knowledge, I am the first to present separate analyses on factors associated with occupation duration for the different nursing occupations, specialized in health and geriatric care.

## Data

I based my empirical analysis on the weakly anonymous Sample of Integrated Labor Market Biographies 1975–2014 (SIAB 7514), a large administrative data set from Germany.^4^ This dataset is based on a 2-percent random sample drawn from the Integrated Employment Biographies (IEB). The IEB contain data on all individuals in Germany who either worked on an employment contract, which is subject to social security contributions (data from 1975 on), or worked under a marginal employment contract (data from 1999 on).^5^ The IEB further contain data on all benefit recipients according to German Social Code III or II legislation (from 1975 and 2005 on, respectively), as well as data on officially registered job seekers and participants in programs of active labor market policies (from 2000 on). The dataset is provided by the Research Data Center of the German Federal Employment Agency (BA) at the Institute for Employment Research.

Data on individual employment episodes are provided by employers in the mandatory process of social security notifications. Data on episodes with other labor market statuses stem from the administrative processes of the BA and the agencies responsible for implementing German Social Code II legislation. The data contain information on employment biographies and a set of individual characteristics as well as characteristics of employment and labor market status, such as an individual’s occupation or the reason for the termination of a period of unemployment benefit reception. The administrative nature of the data collection process results in highly reliable information as far as the respective data are relevant when determining social security payments or the processes of the BA or other data-supplying agencies (e.g. wage and duration of an employment episode). Some variables of less importance to administrative purposes, such as education levels, are less reliable and may contain a considerable number of missing values. Overall, the SIAB 7514 contains over 50 million observations, representing over 1.7 million individuals. The SIAB captures information exact to the day. Information on civil servants, self-employed persons and regular students is not part of the data. See Antoni et al. [4] for detailed information on the SIAB data.

I combine the SIAB data with information on regional characteristics at the county level (*Kreise*). To account for the local labor market situation, local settlement patterns, and local age composition, I augment the data with information on characteristics such as the local unemployment rate, population density, and average age of the population. The respective data stem from BBSR Bonn [6] and the Federal Statistical Office of Germany [62].

For data availability reasons, I employ data from these sources for the years 1998–2010. For employment episodes ending later than November 30, 2011, occupations are reported based on a new occupation classification (Klassifikation der Berufe 2010 (KldB 2010)). As it is not possible to transfer the old classification system into the new one with precision, I do not use episodes beginning after 2010. Data on relevant regional characteristics are only available as of 1998. Contrary to previous studies (e.g. [44] or [33], which employed stock sampling), I employ a flow sample. This means that the sample comprises individuals who entered the analyzed state during a particular interval, which avoids oversampling of longer spells [19]. The flow sample covers the years 1998–2009, with censoring at the end of 2010.

I undertook several steps of data preparation. To correct for missing or potentially misreported values in the administrative education variable, I employed the imputation procedure described in Fitzenberger et al. [31]. The SIAB contains information on employment episodes subject to social security contributions and marginal employment episodes. However, marginal employment is not documented in the data prior to April 1, 1999. To ensure consistency regarding the covered employment episodes without losing all observations older than April 1, 1999, I dropped all marginal employment spells from the data when defining occupational episodes. Because the East German health care system was organized differently from the West German one until 1990 and because the East German labor market for care workers was in a different state than the West German one in the mid-1990s, I focus on West German employment spells. I exclude vocational training spells from the data, as a large share of the geriatric nurses in Germany receive training in vocational schools rather than in establishments. Such training episodes are not part of the SIAB data. I am also interested in the leaving behavior of skilled employees in care occupations who supply labor in tight labor markets, and not in the supply and survival of apprentices. I only analyze spells of persons whose educational level is at least as high as vocational training. Persons over the age of 50 might leave their occupation because of early retirement or due to permanent health issues (a known problem in care occupations) more often than younger people. As my interest lies in nurses’ early exits from their occupation, I exclude spells for persons older than 50 years from the data.

Other than survey data, the SIAB data are highly reliable because of their administrative nature. As a random sample drawn from the IEB, the SIAB as a whole is not prone to problems of sample selection or attrition. To my knowledge, no German dataset other than the SIAB covers such an extensive part of an individual’s labor market biography with such precision. The information on the episodes of employment biographies, as well as their duration, have particularly high precision because of their administrative relevance. Therefore, the data are especially well suited for job and duration analysis, and have already been used by several authors for that purpose [5, 12, 34, 35].

## Econometric methods

To analyze the interdependences among personal, regional, and firm characteristics, changes in macroeconomic and political surroundings, and occupation durations, I apply the piecewise constant mixed proportional hazards model [19]. The probability of leaving work or an occupation is, among others, a function of time elapsed since taking up the occupation. Accounting for a time dimension in standard, e.g. linear or bivariate, regression models is especially problematic as duration data is typically not normally distributed and often, as is the case here, contains right-censored observations. Standard estimation techniques would lead to inefficient or even biased estimates in this case. Duration methods are especially suited in such cases as they account for time dependencies either in offering methods to abstract estimation from time elapsed or by directly modelling such relation. They further handle right-censored observations naturally [47, 20].

For a piecewise constant mixed proportional hazards model, the conditional hazard rate $\lambda \left({\varvec{x}}\right)$ takes the form

$$\lambda \left({\varvec{x}}\right)= {\lambda }_{0}\left(t,\boldsymbol{ }\boldsymbol{\alpha }\right)\phi \left({\varvec{x}}, \, {\varvec{\beta}}\right)\nu , \nu >0,$$

where the baseline hazard ${\lambda }_{0}\left(t, \boldsymbol{\alpha }\right)$ is a step function with $k$ steps, such that

$${\lambda }_{0}\left(t,\boldsymbol{\alpha }\right)={e}^{{\alpha }_{j}}, {c}_{j-1}\le t<{c}_{j}, \, j=1, \dots , k$$

where ${c}_{0}=0$, ${c}_{k}= \infty$, and ${c}_{1}, \dots , {c}_{k-1}$ are additional breakpoints. From breakpoint to breakpoint, the baseline hazard rate remains constant. Parameters $\alpha$ determine the magnitude of the respective baseline hazards and are estimated with the rest of the parameters from the data. Though I can account for a set of individual characteristics by incorporating them in my estimation model directly, an a priory unknown amount of unobserved or unobservable characteristics may influence the individual decision to leave. This can bias my estimation results even if such unobserved heterogeneity was uncorrelated with the regressors [19, 47]. I account for such heterogeneity by introducing a multiplicative heterogeneity term into my model. $\nu$ represents the unobserved heterogeneity. As is proposed by Abbring and van den Berg [1], I assume that $\nu$ is gamma distributed with mean one and variance $\theta$ which can be estimated from the data. The alternative approach, to assume $\nu$ follows an inverse Gaussian distribution, leads to similar estimation results (see Table 1 and Table 8 in the appendix).

To ensure flexibility as well as estimability of the model, I split analysis time into intervals of 28 days during the first year of an employment episode. After the first year, I split analysis time every 60 days. From the third year on, I split analysis time every 180 days. In that way, the model remains reasonably flexible without becoming too extensive. As failure events occur to a decreasing and more stable amount the more time elapsed, it seems sensible to split at shorter intervals at the beginning than later.^6^

**Fig. 1:**
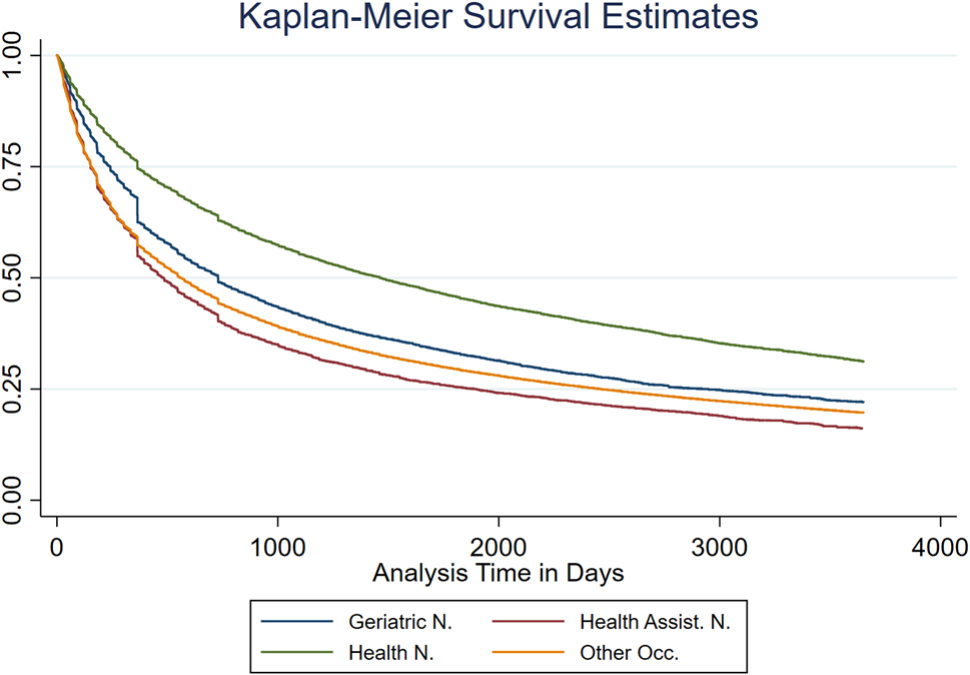
Kaplan–Meier Estimates by Occupations. *Geriatric N* geriatric nurses, *Health Assist. N.*  health nursing assistants, *Health N.* examined health nurses, Other occ. = all other occupations. Source: Own figure based on data of the SIAB 7514

**Table 1:** Results from piecewise constant mixed proportional hazards estimation of occupation duration Source: Own calculations based on data of the SIAB 7514, BBSR [6], Statistisches Bundesamt [62]

Variable		Single risk estimation		Competing risks estimation target states							
		(1)		(2)		(3)		(4)		(5)	
		Single risk		Unemployment		Other occupation		Health & social occupation		Non-health & social occupation	
		hazard ratio	z-value	Hazard ratio	z-value	hazard ratio	z-value	Hazard ratio	z-value	Hazard ratio	z-value
Individual-level characteristics											
Wage (ref. 1st quartile)											
2nd quartile		0.63***	− 20.72	0.60***	− 14.23	0.71***	− 7.47	1.02	0.33	0.44***	− 11.38
3rd quartile		0.37***	− 31.62	0.33***	− 20.47	0.45***	− 12.46	0.69***	− 4.50	0.24***	− 13.06
4th quartile		0.37***	− 22.52	0.34***	− 14.26	0.44***	− 8.96	0.71***	− 3.06	0.21***	− 9.34
Age (ref. 18–30)											
31–40		0.73***	− 12.93	0.85***	− 3.99	0.67***	− 7.92	0.73***	− 4.65	0.61***	− 6.38
41–50		0.60***	− 18.71	0.78***	− 5.81	0.62***	− 8.80	0.65***	− 6.04	0.59***	− 6.18
German citizen		0.97	− 0.84	0.90*	− 1.68	1.33***	3.07	1.35**	2.46	1.29*	1.82
Sex and volume of work (ref. male, full-time)											
Male, part-time		0.88**	− 2.69	0.88*	− 1.79	0.81**	− 2.14	0.87	− 1.20	0.68***	− 2.93
Female, full-time		1.05	1.58	0.92*	− 1.67	1.01	0.18	1.31***	3.36	0.71***	− 3.70
Female, part-time		0.67***	− 11.50	0.59***	− 9.77	0.66***	− 6.17	0.80**	− 2.42	0.51***	− 6.67
Occupation (ref. geriatric nurse)											
Health nurse		0.86*	− 1.64	0.85	− 1.09	0.89	− 0.66	0.83	− 0.79	0.99	− 0.02
Health nurse assist		1.50***	4.65	1.60***	3.50	1.68***	3.03	1.57**	2.04	1.79**	2.15
Firm-level characteristics											
Institution (ref. hospital)											
Nursing homes		1.28***	6.97	1.49***	6.58	2.11***	9.75	2.71***	9.51	1.51***	3.68
Health sector, rest		1.11***	2.33	1.25***	2.88	1.53***	4.40	1.72***	4.06	1.34**	2.06
Social sector, rest		1.22***	5.55	1.46***	6.16	1.77***	7.25	2.25***	7.50	1.32**	2.37
Firm size (ref. < 20 employees)											
20–199		0.82***	− 6.80	0.87***	− 2.91	0.69***	− 6.66	0.60***	− 7.60	0.88	−1.32
Over 199		0.71***	− 8.58	0.67***	− 6.21	0.53***	− 8.05	0.38***	− 9.27	0.84	−1.46
Regional characteristics											
Population density		1.01***	7.89	1.00**	2.10	1.01***	6.16	1.01***	4.17	1.02***	4.59
Unemployment rate		0.98***	− 3.27	1.01	1.50	0.97***	− 2.68	0.98*	− 1.65	0.96**	−2.28
Labor market biography											
Experience in previous 6 years (ref. 3–6 years in care)											
Vocational training		0.84***	− 3.47	0.96	− 0.47	1.07	0.68	1.12	0.86	1.09	0.50
No care experience and											
Other exp	Unemp										
No	No	1.08	1.19	1.61***	4.15	1.42**	2.67	1.23	1.23	1.86***	3.04
Yes	No	1.15***	3.68	1.69***	7.62	1.36***	3.78	1.07	0.68	1.94***	5.21
No	Yes	1.27***	3.64	2.45***	8.96	1.23	1.50	1.13	0.69	1.48*	1.76
Yes	Yes	1.29***	6.79	2.60***	14.76	1.27***	3.01	0.95	-0.47	1.95***	5.30
Up to 3 years of experience in care and											
Other exp	Unemp										
No	No	0.99	− 0.28	1.16*	1.91	1.30***	3.08	1.14	1.18	1.63***	3.63
Yes	No	1.19***	4.09	1.52***	5.41	1.52***	4.95	1.20*	1.62	2.19***	5.92
No	Yes	1.32***	6.06	2.36***	11.35	1.28***	2.57	1.30**	2.23	1.28	1.42
Yes	Yes	1.24***	5.54	2.23***	11.89	1.23***	2.52	1.15	1.38	1.48***	2.87
3 to 6 years of experience in care and											
Other exp	Unemp										
Yes	No	1.09	1.62	1.39***	3.40	1.31***	2.60	1.21	1.43	1.57***	2.65
No	Yes	1.33***	5.65	2.06***	8.63	1.49***	3.99	1.46***	3.18	1.46**	2.06
Yes	Yes	1.38***	5.14	2.22***	8.01	1.38***	2.61	1.21	1.21	1.82***	2.96
Employment episodes (ref. 1)											
2		1.04	1.17	0.98	− 0.34	1.06	0.89	1.09	1.09	1.00	0.02
3		0.99	− 0.22	1.00	0.04	1.10	1.45	1.10	1.02	1.11	0.99
4		1.07*	1.82	1.06	0.94	1.27***	3.09	1.25**	2.23	1.27**	2.09
5		1.18***	3.75	1.29***	3.67	1.28***	2.73	1.24*	1.81	1.32**	1.99
6		1.50***	7.60	1.59***	5.73	1.81***	5.78	2.02***	5.35	1.49**	2.41
7 and more		1.66***	10.11	1.75***	7.50	1.80***	6.03	1.59***	3.56	2.17***	5.34
Further controls											
Year		X		X		X		X		X	
Federal states		X		X		X		X		X	
Time pieces		X		X		X		X		X	
Constant		0.00***	−57.42	0.00***	− 43.46	0.00***	− 36.67	0.00***	− 30.96	0.00***	−24.77
Theta		0.21		0.47		0.55		0.76		1.00	
Subjects		18,325		18,325		18,325		18,325		18,325	

*/**/***  significant at the 10/5/1% level. Experience during previous 6 years: “*other exp.*  employment in other occupation during the last six years; and “*unemp.*  unemployed at some point during the last six years.

The influence of the variables in my model on the risk of leaving care may differ with respect to different target states or different reasons for leaving the occupation. To account for this, I further differentiate between two kinds of models.

First, I analyze individuals leaving nursing occupations regardless of what they do after leaving using single-risk duration models. The dependent variable I analyze is occupation duration, which is the time that elapses after a person enters an occupation until she either leaves it or until her spell is censored due to lack of further information. In my analysis, an individual enters an occupation (becomes “at risk”) by taking up employment in a new job and a new occupation. I count reentry into a previously held occupation as new occupation if the gap between the last and the present employment in that occupation amounts to 90 days or more.^7^ Conversely, an individual leaves the risk set if she leaves an occupation for more than 90 days (after this period, she could become at risk anew). An individual leaves her occupation (the failure event) if she leaves the risk set and the last episode in the risk set contains one of the following employer notifications: end of employment, break of employment following reception of compensation benefits, or break of employment because of parental leave. Spells are censored if an individual leaves the risk set without exhibiting a failure. The maximum length of an episode is 1825 days, meaning that longer episodes are censored after 1825 days. After December 31, 2010, all remaining spells are censored.^8^

In a second set of models, I employ a competing risks framework. Here, the conditional hazard rate becomes

$${\lambda }_{j}\left({\varvec{x}}\right)= {\lambda }_{0j}\left(t, \boldsymbol{\alpha }\right)\phi \left({\varvec{x}},{\varvec{\beta}}\right){\nu }_{j},$$

and is now specific to hazard of type $j$. The different types of hazard I consider here are transition into unemployment, transition into employment in a different occupation, and finer-grained employment in another occupation in or outside the domain of health and social occupations. This is motivated by the fact that for health policy as well as for the welfare state, it is not only interesting to know what generally drives nurses out of their occupation, but also what drives them to these different target states. Also, analyses of leaving behavior with respect to different target states provide hints regarding the mechanisms that drive leaving behavior and the respective routes. I assume that hazards from the different types of risks are uncorrelated (independent risks assumption). Though it is not unrealistic that certain factors might influence leaving behavior into the different target states, and that these factors may be correlated, I minimize the influence of those uncontrolled dependencies by using an extensive number of variables covering possible differences among individuals at the firm and county levels. One specific source of concern may be individual preferences. Individual preferences for work may increase the tendency to leave for another type of employment and decrease the tendency to leave for unemployment. The tendency to leave for an occupation outside the health care and social domain may be associated with preferences for occupational mobility in the same way. To cope with such differences in individual preferences, I account for individuals’ number of prior occupation episodes as well as labor market experience in the six years prior to the present employment episode, and include whether an individual worked outside the care industry or has been unemployed. I estimate the competing risks framework by estimating separate models for each kind of risk [19].

To differentiate between different starting conditions over individuals, I account for the labor market biography of an individual up to six years prior to entering their occupation under analysis. I differentiate between those who have gathered no experience in care in the last six years, those who have been employed in a care occupation up to 3 years, and those who have been employed in care occupations for 3–6 years. For all of those, I further differentiate individuals according to whether they had been unemployed during the last six years or whether they had been employed in a non-care occupation during that time.

I include several individual-level, firm-level, and county-level characteristics in the proportional hazard models. On the individual level, I include demographic characteristics (age group, sex, level of education, German or non-German nationality) and occupational characteristics (occupation, part-time or full-time employment, daily wages, and the individual’s previous labor market biography). On the firm level, I include firm size groups measured via the number of employees; location of the firm by federal state; and a categorical variable indicating whether the firm is a hospital, a nursing home, operates in the rest of the healthcare sector, or the rest of the social sector. On the county level, I employ demographic information (population density), as well as economic information (unemployment rate). Table 5 in the appendix summarizes the variables and their definitions.

## Results

### Univariate analysis

Figure 1 contains Kaplan–Meier survival functions, which offer a descriptive overview of occupation durations for examined health nurses (Health N.), geriatric nurses (Geriatric N.), health nursing assistants (Health Assist. N.), and all other occupations (Other occ.). Among all groups, individuals leave their occupation more often at the beginning of a new occupation episode, with especially high numbers leaving at the end of the first and the second year. The groups clearly differ in the number of individuals leaving their occupation. Health nursing assistants leave the fastest and are the only nursing occupation to leave faster than the group of all other occupations. Geriatric nurses leave their occupation almost as often as the group of all other occupations, and examined health nurses leave their occupation the least often. Overall, nurses do not seem to leave faster than the average compared to all other occupations.

### Multivariate analysis

Figure 1 hints at the leaving behavior of the different nursing occupations and their leaving behavior compared to that of individuals working in other occupations. In the following, I further separate factors associated with a higher or lower tendency to leave one’s occupation for individuals in nursing occupations, using proportional hazards models.

Column [1] of Table 1 displays estimation results for a piecewise constant mixed proportional hazards model. Table 7 in the appendix shows results for a Cox proportional hazards model. The models refer to examined hospital nurses, geriatric nurses, and hospital nursing assistants. I further differentiate the estimations with regard to the different occupations (see Table 9 in the appendix).

### Basic individual characteristics

Most of the estimated coefficients are comparable in direction and size among the separate models, meaning they are relatively robust to changes in estimation approach. The results for the time pieces which account for the time elapsed since an individual (re)entered a care occupation (reported in Table 6 and Figure 5 in the appendix) exhibit a decreasing pattern until half a year after entry, a point in time which is covered by the time piece of 168 to 196 days. Afterwards the baseline hazard stays relatively constant. The time pieces including half a year, a year, and two years’ time since entry deviate from this pattern, at these points leaving is especially likely.^9^ The latter finding shows that nurses often leave after fixed intervals. This may be because employment contracts in Germany can often only be terminated at fixed times, such as the end of a quarter. The decreasing hazard over time and the fact that the hazard also decreases with age is in line with job search literature and a large amount of empirical literature on job changes. Namely, younger nurses and nurses who just (re)entered the occupation leave it more often [18, 48, see 30 for a relevant overview]. These results show that younger nurses and nurses who just (re)entered care should receive special attention in policies aimed at longer occupation duration of nurses, especially at the end of the first half and full year as well as the second year in the occupation. I find no statistically significant association between non-German nationality and leaving behavior.

### Child rearing and work arrangements

A vast majority of workers in care occupations are female (see, e.g. Drennan and Ross [27], the numbers in Table 5—approximately 82 percent—are close to those Bogai [10] reports for Germany). As women usually bear the lion’s share of care work for children within their families,^10^ the question arises whether having children increases women’s tendencies to leave the occupation or whether they can combine care occupation employment and child rearing. Part-time work arrangements can be one way to do so. My data does not contain direct information on whether individuals have children. However, Müller and Strauch [52] show a way to identify childbirth events for female workers in the SIAB data. Regarding women who receive social security benefits or who are seeking a job, they identify childbirth via the respective deregistration reports of “maternity benefit” or “maternity leave” in the data. Regarding employed women, they identify childbirth if women below age 40 (38 for the first child) exhibit an employment interruption “because of entitlement to other compensation,” for which the interruption has to last for at least 98 days (the duration of maternity leave), as that same interruption notification can also indicate long-term sickness. I use their approach to identify mothers and the age of their children in my data.

In line with Doiron and Jones [26], Nooney et al. [53], and Rongen et al. [57], I find women leave nursing less often than men do (see column [1] of Table 10 in the appendix). When I further account for whether women and men work part-time or full-time, I find men and women leave less often when employed part-time (see column [1] of Table 1). The effect is stronger for women, however. I further differentiate according to whether women have children younger than 14. With 53 percent, the decrease in the hazard rate for women with children is more than two times as large as the effect for women without children (see Table 10 in the appendix). Therefore, part-time work seems to be a strategy female nurses use to align professional work and care work within their families. Providing more part-time positions may be a solution to keep more female nurses in the occupation. Female nurses may stay longer in a care profession if employed part-time, even if they do not have children, because of further unpaid care work within the family (e.g. for the elderly) or other reasons that I cannot identify (e.g. cultural values or health issues).

### Job characteristics

Further, I find clear associations between several job factors and occupation duration. Nurses employed in the health sector leave their occupation less often than nurses employed in nursing homes or the rest of the social sector do. Nurses employed in hospitals leave their occupation the least often. Moreover, even after controlling for sectors, nurses who work with a larger employer (e.g. large hospitals, such as university hospitals) leave their occupation less often than nurses who work with smaller employers (e.g. small county hospitals) do. The association between an institution’s size and leaving hazard exhibits different patterns across countries. For example, Holmås [44] finds hazard rates increase for larger institutions, such as university hospitals, in Norway, while Frijters et al. [33] and Doiron and Jones [26] find the reversed effect for NHS nurses in the UK and nurses in Australia, respectively. The finding that nurses leave slower when their employer is larger is in line with what has previously been found for the job duration in the overall German labor market [12, 34, 35, examining work in temporary help agencies, only: 5]. Explanations for this pattern could be sorting processes, where more motivated nurses sort into bigger, and perhaps more specialized institutions, such as university hospitals, or in organizational features of larger institutions, which may offer more career options within the nursing occupation.

I also find a significant negative effect of daily wages on the propensity to leave nursing. Wages enter my model in the form of the four wage quartiles. Higher daily wages associate with a strong decrease in the probability of leaving nursing. The effect increases for the first, second, and third quartiles. However, the hazard ratio for the third wage quartile is the same as the one for the fourth quartile. Wage raises, therefore, may be an effective way to increase nurses’ retention rates. The effect of higher wages seems to wear off at some point, however.^11^

### Outside options

Furthermore, the availability of alternative job or occupational options on the regional labor market seems to drive the leaving decision. I approximate those options via local population density and unemployment. Nurses in more densely populated areas or areas where unemployment is lower leave their occupation more often; employees should have more alternative options in flourishing labor markets in densely populated regions [48].

### Labor market biographies

**Fig. 2:**
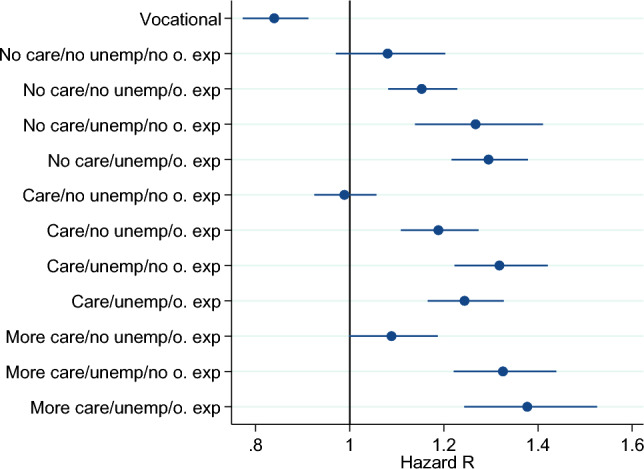
Estimates for Groups with Different Experiences Before Entry. Dots indicate point estimates converted into hazard ratios, and blue lines indicate the 90 percent confidence interval around the point estimate. A group’s labor market experience is indicated by whether they had experience in care during the previous six years (no care: no care experience; care: up to three years of experience; and more care: three to six years of experience during the past six years), whether they have been unemployed in the previous six years (no unemp: no unemployment, unemp: unemployed at some point during the last six years) and whether they have been employed in another occupation during the previous six years (no o. exp: no employment in other occupation; o. exp: employment in other occupation during the last six years). Those who just entered out of vocational training build the group vocational. Base category is no prior unemployment, no experience in another occupation and three to six years of experience in care during the last six years. Source: Own figure based on data of the SIAB 7514

Leaving behavior also differs with labor market biographies. Differentiating groups of nurses according to their prior labor market biographies, their experience in and out of care, and unemployment during the six years before they entered their present occupation episode in care, I find striking differences. I give the estimates for groups of care personnel with different experience among the other estimation results in the tables. However, for a handier representation of the respective estimates, Fig. 2 provides graphical representations of the coefficient estimates and the respective 95 percent confidence intervals. I differentiate the groups along three dimensions: (1) whether a person has been unemployed during the last 6 years (yes or no); (2) whether she worked in a non-care occupation during the last six years (yes or no); and (3) how long an individual worked in a care occupation in the prior six years (no exp. = not at all; some exp. = until 3 years; and more exp. = between 3 and 6 years). Table 4 in the appendix gives a detailed definition of the respective groups. The figure shows, first, care personnel leave their occupation more often if they had been employed with a non-care occupation in the six years before they entered their present occupation. Second, the same holds true for those nurses who were previously unemployed. Last, nurses who entered their present occupation episode directly after occupational training leave the least often. Regarding the amount of experience a nurse gained in care occupations during the six years prior to the present episode, I find no apparent effect on occupation duration. A nurse’s occupation duration, therefore, is not associated with prior experience in nursing. Either nurses do not accumulate significant human capital *in the occupation*, meaning human capital accumulation predominantly happens during nurses’ occupational training, or the human capital nurses accumulate while working in nursing is rather job-specific than occupation-specific.

To account for an individual’s overall tendency to switch occupations, I incorporate into the estimation model the number of prior occupation episodes a person exhibited before becoming at risk. In line with previous literature, I find the higher the number of prior occupation episodes, the higher the tendency to leave the present occupation. Therefore, the number of prior episodes indeed differentiates between those more inclined and those less inclined to switch—a measure of the respective heterogeneity, which Farber [30] also discussed and Farber [29] employed. Further, the number of prior episodes has recently been shown to be a signal for different work attitudes [21].

### Competing risks

From the viewpoint of health policy and the welfare state, it is not only interesting to know what drives nurses out of their occupation but also what drives them into different target states. Nurses leaving into unemployment are a larger issue for the welfare state than those leaving for another occupation. Nurses leaving for another occupation in the health and social domain may seem less problematic than those who leave for a completely different occupation. In addition, analyses of leaving behavior, with respect to different target states, provide hints regarding the mechanisms that drive leaving behavior and the respective routes. Therefore, I differentiate the association between labor market biographies and leaving behavior over different target states–unemployment, employment in another occupation, employment in an occupation in the spectrum of health and social occupations, and employment in an occupation outside the spectrum of health and social occupations. Columns [2] to [5] of Table 1 display results of competing risks estimations regarding the respective target states. Regarding the target state of unemployment, previous unemployment experience is a powerful indicator of an exit into unemployment. However, previous experience in other occupations also has a positive effect on the tendency to become unemployed, though not as large as the effect of previous unemployment. More experience in care diminishes the hazard of leaving into unemployment. Regarding the tendency to leave for another occupation, I find a positive association between previous experience in another occupation and the hazard of leaving. However, I also find a positive association between previous unemployment experience and the hazard rate.

There are also differences regarding other explanatory variables with regard to the respective target states. Nurses of German nationality have a lower propensity to leave for unemployment. Germans leave more often for other occupations, in general, and for other health or social occupations, in particular, than their non-German peers do. Wage is relevant in all leaving scenarios and the sizes of the effects of earing wages in one of the wage quartiles are comparable to the single risks estimation results for the target states of unemployment and employment in any other occupation. Differences show, however, in statistical significance and effect size regarding the propensity to leave to another occupation in the health and social sector or to an occupation outside this occupational sector. The tendency to leave to an occupation outside the health and social domain decreases considerably stronger with rising wages than the tendency to leave to an occupation inside the health and social sector. What is more, small differences in wages have a significant effect in the former case but not in the latter. Wage increases, even smaller ones, may therefore be a way to keep nurses from leaving the health and social occupations altogether, even if larger wage increases would be needed to keep them from leaving to another occupation in the health and social domain.

I find two other striking patterns regarding different associations of local characteristics and leaving behavior differentiated by target states. As noted, nurses leave their occupation less often in counties where unemployment is higher. The picture becomes more nuanced when I differentiate between target states. On the one hand, I do not find a significant effect of the local unemployment rate on leaving into unemployment. On the other hand, where the unemployment rate is higher, nurses are less likely to leave for another occupation. This is sensible because other occupational options are scarcer where the local unemployment rate is higher. Therefore, nurses have worse chances to find employment in another occupation if they want to leave nursing. The other pattern, in which nurses leave for other occupations more often where population densities are higher, is also sensible. The more numerous and better the alternative occupational options, the higher the population density, and the higher the probability that a nurse takes one of those options. Overall, the competing risk analyses support the argument that nurses leave more often in areas where the unemployment rate is lower or the population density is higher, due to more abundant occupational alternatives. However, this means that care work would have to become more attractive relative to other employment to keep nurses from leaving for other occupations in counties where they have the possibility.

### Heterogeneity among different care occupations

Care personnel in Germany, other than in other countries, have long been divided into different groups according to their main area of action and their level of occupational training. Geriatric nurses mainly work with elderly people, and hospital nurses mainly work in caring for the ill. Further, geriatric and hospital nurses may have the occupational education to work as a registered geriatric or hospital nurse or as a geriatric or hospital nursing assistant. This separation along areas of care and level of occupational education is grounded in the history and education system of care in Germany [10].

In my main model, I introduce an indicator variable for the different occupations I can differentiate in the data: one group is registered hospital nurses; another group is made up from hospital nursing assistants; and the last group comprises registered geriatric nurses and geriatric nursing assistants, as I cannot differentiate between these two groups in my data. In line with the results of the Kaplan–Meier estimation, hospital nursing assistants leave their occupation significantly more often than nurses in the other occupations do, even after controlling for other factors. This seems at odds with Frijters et al. [33], who find registered nurses and nurses in higher positions leave (in their case, the NHS) more often. On the one hand, this could be based on the different leave events with which Frijters et al. [33] deal (leaving NHS rather than leaving the occupation) or the different organization of the German occupational system, for which registered nurses need to complete a three-year training and nursing assistants a one-year training. Therefore, registered nurses accumulate a significantly higher amount of occupation-specific human capital—which also indicates considerably higher wages [11]—and which may leave them less inclined to leave their occupation. This is also sensible, as hospital nursing assistants are more inclined to leave nursing for an occupation outside the health and social domain than registered hospital nurses are, as can be seen in Table 2 and column [5] of Table 1. Further, registered hospital nurses leave nursing more often than geriatric nurses do. The effect is less clear, however. Differences in the tendency to leave between geriatric nurses and hospital nurses, as I found in the Kaplan–Meier estimation, are explained, at least in part, by other factors in my multivariate model. Notably, I get a much stronger effect for registered hospital nurses when excluding the sector variable from the model (not reported). This indicates that a reason for the lower propensity of registered health nurses to leave their occupation is that they would rather work in the health sector than in the social or any other sector, whereas geriatric nurses work in the social sector more often. The reason for the higher propensity for geriatric nurses to leave their occupation that I find in the Kaplan–Meier estimation, therefore, is partly explained by the sector in which they work, rather than in their respective occupation.

**Table 2:** Occupational status up to 1 year after leaving the occupation Source: Own calculations based on data of the SIAB 7514

	Geriatric nurse	Registered hospital nurse	Hospital assistant nurse	All nursing occupations
Unemployed	28%	15%	28%	23%
Vocational Training	2%	1%	3%	2%
Last Episode	9%	14%	8%	11%
Employment subject to social security contributions				
Outside health occupations	10%	7%	11%	9%
In health occupation	27%	28%	23%	26%
Marginal employment				
Outside health occupations	3%	1%	4%	3%
In health occupation	5%	7%	6%	6%
Other, not defined	15%	28%	16%	20%
N	5357	6239	3626	15,222

To examine potential differences in leaving behavior between the different groups of nurses, I further estimate a separate model for geriatric nurses (registered and assistants), one for registered hospital nurses, and one for hospital nursing assistants. The results are given in Table 9 in the appendix. The results do not point to remarkable differences between the factors associated with geriatric or hospital nurses’ leaving decisions. However, I find differences with regard to the occupational status nurses exhibit after leaving their occupation. Most of the nurses, for which I can define clear post-leaving states, leave into unemployment, another health occupation, or another occupation outside the health sector, where it is noticeable that examined hospital nurses end up unemployed to a lower amount and employed in another health occupation to a higher amount than individuals from the other nursing occupations are. This may be because of the relatively higher overall education level of examined hospital nurses and better possibilities to transfer their human capital from hospital nursing to other health occupations. Table 2 reports the target states of nurses who left the occupation.

### Changes in occupation-specific regulations

**Fig. 3:**
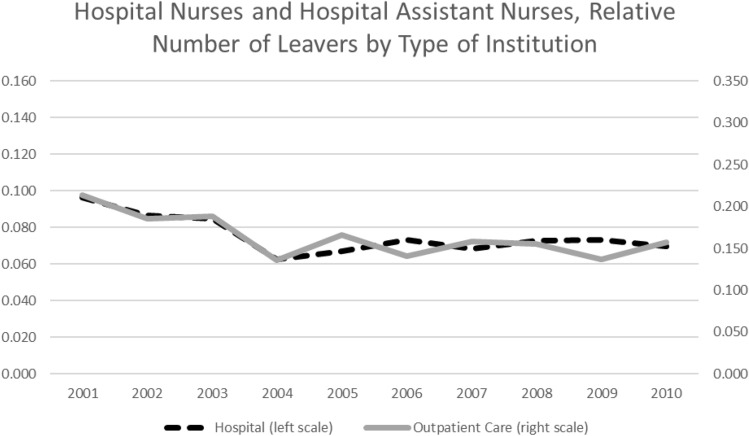
Annual Share of Nurses Leaving Relative to Their Respective Totals by Institution. Source: Own figure based on data of the SIAB 7514

During the analyzed period, at least two relevant institutional changes occurred, which could have had an influence on nurses’ occupation durations. For one thing, the introduction of diagnosis-related groups (DRG) in the reimbursement system of hospital services in Germany. In addition, Germany introduced a nursing minimum wage. The new reimbursement system was first introduced on a voluntary basis in 2003 and became mandatory in 2004. The system brought a new regulation for the reimbursement of hospital services based on diagnosis-based base rates. Since its introduction, the German DRG system has been suspected to worsen working conditions among hospital personnel by way of an “economization” of hospital care, thereby fostering nurses’ departures from the occupation [13, 14, based on studies regarding the introduction of the DRG system in the United States: 56]. The DRG system only deals with the reimbursement of hospital services. Hence, if the new DRG system actually raised the number of nurses leaving care, only nurses employed in hospitals should been affected. Therefore, the number of nurses leaving hospital nursing should have increased relative to the number of nurses leaving nursing outside a hospital framework after the DRG introduction. Figure 3 shows the number of health nurses and health nurse auxiliaries departing from care in a hospital framework relative to all nurses employed in hospitals (dashed black line), as well as the number of nurses leaving nursing who are employed in outpatient care (solid grey line) on a yearly basis. With few exceptions, the two lines move simultaneously over the relevant time interval—nothing hints at an increased leaving hazard for nurses in hospitals. This stays true in a multivariate context.^12^ This could be a challenge to the thesis that nurses’ working conditions in the hospital context worsened after introducing the DRG system in Germany. Otherwise, it could be the case that the working conditions did not worsen enough to drive a significant number of nurses out of the hospitals.

**Fig. 4:**
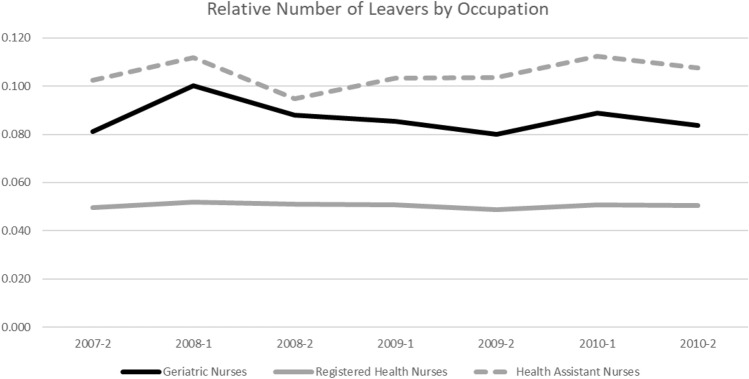
Semiannual Share of Nurses Leaving Relative to Their Respective Totals by Occupation. Source: Own figure based on data of the SIAB 7514

**Fig. 5:**
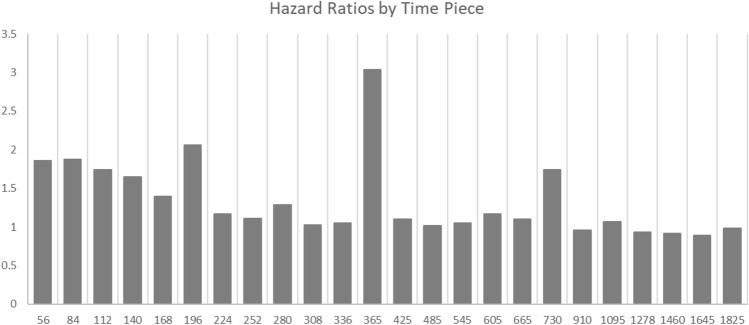
Results from proportional hazards estimation, hazard ratios for time pieces. Source: Own figure based on data of the SIAB 7514

I find a similar result regarding the introduction of the nursing minimum wage. Figure 4 shows the number of geriatric nurses (solid black line), registered health nurses (solid grey line), and health nurse auxiliaries (dashed grey line) leaving their occupations relative to all nurses in the respective group on a semiannual basis. As assistant nurses receive the lowest pay, those should be the group most affected by the October 2010 introduction of the minimum wage. However, health nurse auxiliaries do not show a distinct downward trend in the relative number of individuals who left the profession. Therefore, introducing the minimum wage does not seem to have had an immediate influence on nurses’ leaving behavior.^13^ At first sight, this seems at odds with economic theory and the results I present for the association between wage and nurses’ occupation durations (i.e. higher wages are associated with longer occupation duration). However, this result is in line with results from previous studies [40, 46]. It seems likely that the introduction of the minimum wage affected only a small share of nurses in western Germany. However, reactions may take place with a longer time lag, for which I cannot account because of changes in reporting schemes in my data.

## Conclusion

In this paper, I study the duration of nurses’ occupation episodes and factors associated with longer or shorter occupation duration.

Other than one might guess in light of the public debate, nursing occupations overall do not exhibit specifically short occupation durations. There is already a skilled labor shortage in nursing, and the number of possible newly educated nurses is unlikely to rise significantly because of demographic changes. Thus, keeping nurses at work as long as possible can be seen as a key strategy for maintaining a sufficient number of nursing staff. I further estimate the impact of different factors on nurses’ decisions to leave their occupation. Independently from the different nursing occupations and care settings (inpatient and outpatient care), younger nurses, nurses in the social sector, nurses working with a smaller employer, and nurses who receive lower wages leave their occupations more often than their respective counterparts do. A considerable share of nurses is employed part-time, and part-time employment decreases the leaving propensity of female nurses, in particular for nurses with children. In addition, nurses leave more often when alternative occupational options are better. Nurses who had been unemployed and nurses who had worked in a different occupation in the past have a higher probability of leaving the occupation, whereas nurses who have just finished vocational training have a lower propensity to leave. I employ competing risks analysis and analyze different target states. To where nurses leave their occupation differs with respect to regional characteristics, individual characteristics, wage, and experience. Finally, I cast a brief look at two institutional changes in the context of nursing in Germany: the introduction of the DRG system and the introduction of the nursing minimum wage. Neither influenced nurses’ occupation durations.

My findings have implications for targeted hiring decisions and efficient ways to retain nurses in their occupations. Where possible, nurses should be hired directly after vocational training, and special effort should be undertaken to prevent breaks in their labor market biographies. Better possibilities to reconcile family commitments and care work could be one option to retain the mostly female care workforce in their occupations as long as possible and to prevent significant breaks in their work biographies. When nurses are hired out of unemployment or after an employment episode in a different occupation, employers could offer special working arrangements and training to ease the (re)entry into the nursing occupation. Where nurses have many professional alternatives, the attractiveness of care work must increase to keep nurses working in their occupation. However, keeping nurses at work as long as possible can only be part of an overall strategy to tackle the nursing labor shortage. Recruitment of new nursing personnel is also vital. One possibility to accomplish this is to recruit care personnel from abroad; another is to bring more domestic school graduates into nursing occupations. Knowledge about the factors determining young persons’ career choice and influencing the decision to enter or not to enter a care profession is vital in this context and the subject of an ongoing scientific debate.

The data I employ offers detailed information on individuals’ labor market biographies and highly reliable information on their employment; however, the data has no detailed information on socioeconomic variables and individuals’ household contexts and family situations. I analyze interdependencies between occupation duration and individual characteristics, as far as possible. A more detailed analysis of the relationship between occupation duration and the respective characteristics remains a task for future research.

## Figures and Tables

**Fig. 6:**
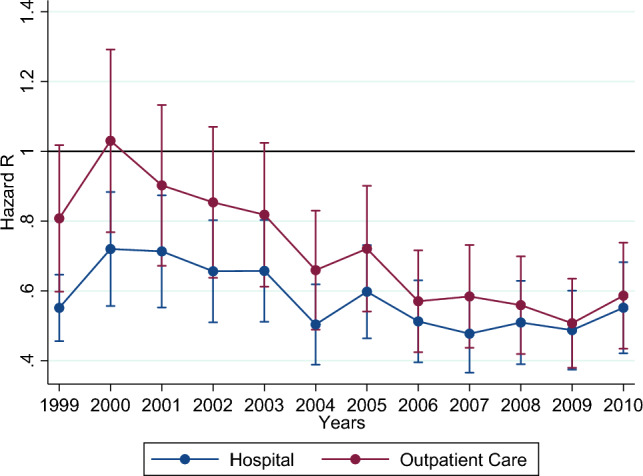
Results from a Multivariate Proportional Hazards Estimation, Hazard Ratios for Years for Nurses Employed in Hospitals and Outpatient Care. Source: Own figure based on data of the SIAB 7514

**Fig. 7:**
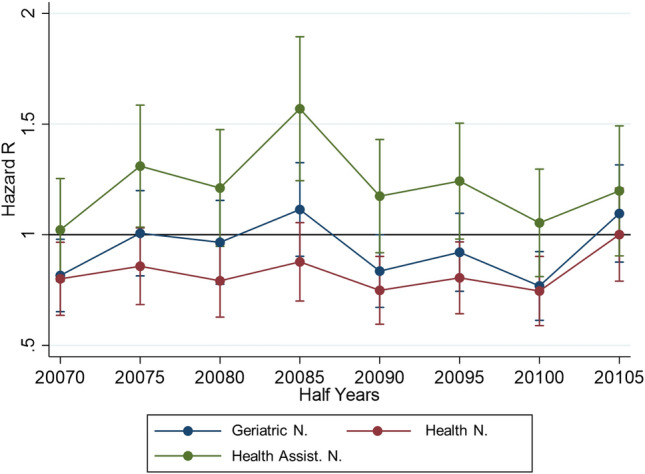
Results from a Multivariate Proportional Hazards Estimation, Hazard Ratios for Years for Nurses Employed in Hospitals and Outpatient Care. *Geriatric N.*  geriatric nurses, *Health Assist. N.*   health nursing assistants, *Health N.* examined health nurses. 20070, 20080, 20090, and 20100 refer to the first half of the years 2007, 2008, 2009, and 2010, respectively. 20075, 20085, 20095, and 20105 refer to the second half of the years 2007, 2008, 2009, and 2010, respectively. Source: Own figure based on data of the SIAB 7514

## Data Availability

The dataset analyzed in the current study is not publicly available due to data protection laws (social security data) but can be accessed on-site at the Institute for Employment Research (IAB) subject to an individual data protection agreement (More details on the structure and origin of the data can be found in the data description by Antoni et al. [4].

## Appendix

See Tables 3, 4, 5, 6, 7, 8, 9, 10, 11 and Figs. 5, 6 and 7

**Table 3:** Definitions of entry, exit, posterior labor market state

Duration in occupation–failure with simultaneous job end, only	
Becoming at risk	Entering a new job and occupation
Leaving risk set	Leaving the occupation without return in the following 90 days or staying in occupation without wage for at least 90 days
Failure	Leaving risk set with End of employment notification by employer (“grund = 30 or 40”) Break due to following reception of compensation benefits notification by employer (“grund = 51”) Break due to following parental leave episode notification by employer (“grund = 52”)
Censored	Leaving risk set without a failure; alternatively: still in risk set on 31 December 2010
Posterior labor market state	
Employed in	
… different health occupation	Worker is employed in a health occupation (other than the current one) in the following episode (one year after current occupation at maximum)
… different occupation, not health	Worker is employed in another occupation (no health occupation) in the following episode (one year after current occupation at maximum)
Unemployed	Worker is unemployed in the following episode
No status or employed without income	No information on the worker’s posterior status, or status is employed with income equal to zero
In vocational training (reported)	Vocational training is reported in the following episode
Last episode	There are no following episodes of the individual covered by the data

The posterior labor market statuses after taking up the respective occupation take into account information up to one year after leaving

**Table 4:** Experience in prior 6 years, definition Source: Own calculations based on data of the SIAB 7514

Variable		Mean	Definition
Vocational training		0.10	Entering after vocational training
No experience in care and			
Other exp	Unemp		
No	No	0.03	Neither registered unemployment nor employment episodes during the 6 prior years
Yes	No	0.11	Experience outside care during the prior 6 years
No	Yes	0.03	Some period of unemployment during the prior 6 years
Yes	Yes	0.12	Some period of unemployment during the prior 6 yearsand experience outside care during the prior 6 years
Up to 3 years experience in care and			
Other Exp	Unemp		
No	No	0.09	Up until 3 years of experience in care during the prior 6 years
Yes	No	0.07	Up until 3 years of experience in care during the prior 6 yearsand experience outside care during the prior 6 years
No	Yes	0.06	Up until 3 years of experience in care during the prior 6 yearsand some period of unemployment during the prior 6 years
Yes	Yes	0.10	Up until 3 years of experience in care during the prior 6 yearsand some period of unemployment during the prior 6 yearsand experience outside care during the prior 6 years
3 to 6 years experience in care and			
Other exp	Unemp		
No	No	0.19	Between 3 and 6 years of experience in care during the prior 6 years – base category
Yes	No	0.05	Between 3 and 6 years of experience in care during the prior 6 year sand experience outside care during the prior 6 years
No	Yes	0.05	Between 3 and 6 years of experience in care during the prior 6 year sand some period of unemployment during the prior 6 years
Yes	Yes	0.03	Between 3 and 6 years of experience in care during the prior 6 year sand some period of unemployment during the prior 6 years and experience outside care during the prior 6 years

**Table 5:** Variables and definitions Source: Own calculations based on data of the SIAB 7514

Variable	Mean	Definition
Individual-level characteristics		
Wage	58.53	Daily wage in euros deflated to 2010 consumer prices
Part-Time	0.44	1 if observation refers to part-time employment episode, 0 if full-time
Age	32.69	Age
Female	0.82	1 if observation refers to female individual, 0 in observation refers to male individual
German citizen	0.94	1 if observation refers to a German citizen, 0 otherwise
Occupation		
Geriatric nurse	0.35	1 if observation refers to a geriatric nurse, 0 otherwise
Health nurse	0.43	1 if observation refers to a health nurse, 0 otherwise
Health nurse assist	0.23	1 if observation refers to a health nurse assistant, 0 otherwise
Firm-level characteristics		
Institution		
Hospital	0.33	1 if observation refers to employment in a hospital, 0 otherwise
Nursing homes	0.36	1 if observation refers to employment in a nursing home, 0 otherwise
Health sector, rest	0.07	1 if observation refers to employment in the rest of the health sector (not hospital), 0 otherwise
Social sector, rest	0.23	1 if observation refers to employment in the rest of the social sector (not nursing home), 0 otherwise
Firm size		
< 20 employees	0.12	1 if observation refers to employment in an establishment with less than 20 employees, 0 otherwise
20–199	0.51	1 if observation refers to employment in an establishment with 20–199 employees, 0 otherwise
Over 199	0.37	1 if observation refers to employment in an establishment with more than 199 employees, 0 otherwise
Regional characteristics		
Population density	9.22	Local population density (100 inhabitants per km^2)
Unemployment rate	8.35	Local unemployment rate in percent
Labor market biography		
Employment Episodes		Running sum of occupation episodes as defined for the analysis
1	0.26	1 if first episode, 0 otherwise
2	0.27	1 if second episode, 0 otherwise
3	0.20	1 if third episode, 0 otherwise
4	0.12	1 if fourth episode, 0 otherwise
5	0.06	1 if fifth episode, 0 otherwise
6	0.04	1 if sixth episode, 0 otherwise
7 and more	0.05	1 if seventh episode or episode of higher count, 0 otherwise

**Table 6:** Results from proportional hazards estimation, hazard ratios for time pieces Source: Own calculations based on data of the SIAB 7514, BBSR [6], Statistisches Bundesamt [63]

Variable	All nurses	
	Piecewise constant proportional hazards model—heterogeneity	
	Hazard ratio	z value
Time pieces: days, up to		
56	1.86***	11.05
84	1.88***	11.09
112	1.74***	9.50
140	1.65***	8.35
168	1.40***	5.34
196	2.06***	12.27
224	1.17**	2.28
252	1.11	1.46
280	1.29***	3.69
308	1.03	0.41
336	1.05	0.69
365	3.04***	19.49
425	1.10	1.50
485	1.02	0.34
545	1.05	0.80
605	1.17**	2.40
665	1.10	1.46
730	1.74***	8.97
910	0.96	− 0.76
1095	1.07	1.10
1278	0.93	− 1.13
1460	0.92	− 1.26
1645	0.89	− 1.55
1825	0.98	− 1.36
Theta	0.21	
N	502,478	
Subjects	18,323	

**Table 7:** Results from proportional hazards estimation without frailty term

Variable		Cox-PH model	
		Hazard ratio	z value
Individual-level characteristics			
Wage (ref. 1st quartile)			
2nd quartile		0.64***	− 20.05
3rd quartile		0.39***	− 29.28
4th quartile		0.40***	− 20.41
Age (ref. 18–30)			
31–40		0.73***	− 13.21
41–50		0.60***	− 18.65
German citizen		0.98	− 0.43
Sex and volume of work (ref. male, part-time)			
Male, part-time		0.89**	− 2.41
Female, full-time		1.05***	1.58
Female, part-time		0.69***	− 10.95
Occupation (ref. geriatric nurse)			
Health nurse		0.89	− 1.29
Health nurse assist		1.49***	4.68
Firm-level characteristics			
Institution (ref. hospital)			
Nursing homes		1.22***	5.92
Health sector, rest		1.11***	2.19
Social sector, rest		1.20***	5.21
Firm Size (ref. < 20 employees)			
20–199		0.82***	− 7.07
Over 199		0.72***	− 8.90
Regional characteristics			
Population density		1.01***	8.65
Unemployment rate		0.98***	− 3.70
Vocational training		0.84***	− 3.74
Other exp	Unemp		
No	No	1.06	0.99
Yes	No	1.13**	3.37
No	Yes	1.24***	3.53
Yes	Yes	1.22***	5.32
Other exp	Unemp		
No	No	1.02	0.45
Yes	No	1.21***	4.53
No	Yes	1.36***	6.83
Yes	Yes	1.28***	6.44
Other exp	Unemp		
Yes	No	1.07	1.35
No	Yes	1.33***	5.91
Yes	yes	1.38***	5.10
Employment Episodes (ref. 1)			
2		1.05*	1.70
3		1.01	0.37
4		1.11***	2.78
5		1.20***	4.15
6		1.53***	8.18
7 and more		1.75***	11.15
Further controls			
Year		X	
Federal states		X	
Time pieces		–	
Constant		–	
Theta		–	
Subjects		18,317	

*/**/***  significant at the 10/5/1% level

Standard errors are clustered at individual level

Experience in previous 6 years. “*other exp.*”: employment in other occupation during the last six years, *unemp.*: unemployed at some point during the last six years. Source: Own calculations based on data of the SIAB 7514, BBSR [6], Statistisches Bundesamt [62]

**Table 8:** Results from proportional hazards estimation with inverse gaussian frailty term

Variable		Single risk estimation	
		Hazard ratio	z value
Individual-level characteristics			
Wage (ref. 1st quartile)			
2nd quartile		0.62***	− 20.74
3rd quartile		0.36***	− 31.63
4th quartile		0.37***	− 22.54
Age (ref. 18–30)			
31–40		0.73***	− 12.96
41–50		0.59***	− 18.73
German citizen		0.96	− 0.88
Sex and volume of work (ref. male, full-time)			
Male, part-time		0.88**	− 2.71
Female, full-time		1.05	1.56
Female, part-time		0.67***	− 11.47
Occupation (ref. geriatric nurse)			
Health nurse		0.86*	− 1.63
Health nurse assist		1.51***	4.64
Firm-level characteristics			
Institution (ref. hospital)			
Nursing homes		1.28***	6.95
Health sector, rest		1.11**	2.32
Social sector, rest		1.22***	5.54
Firm size (ref. < 20 employees)			
20–199		0.82***	− 6.81
Over 199		0.71***	− 8.62
Regional characteristics			
Population density		1.01***	7.87
Unemployment rate		0.98***	− 3.26
Labor market biography			
Vocational training		0.84***	− 3.49
Other exp	Unemp		
No	No	1.08	1.15
Yes	No	1.15***	3.67
No	Yes	1.26***	3.59
Yes	Yes	1.30***	6.78
Other exp	Unemp		
No	No	0.98	− 0.38
Yes	No	1.19***	4.03
No	Yes	1.31***	5.86
Yes	Yes	1.24***	5.46
Other exp	Unemp		
Yes	No	1.09*	1.65
No	Yes	1.33***	5.65
Yes	Yes	1.38***	5.14
2		1.04	1.17
3		0.99	− 0.23
4		1.07*	1.76
5		1.18***	3.63
6		1.49***	7.44
7 and more		1.64***	9.71
Further controls			
Year		X	
Federal states		X	
Time pieces		X	
Constant		0.00***	− 57.11
Theta		0.26	
Subjects		18,325	

*/**/***  significant at the 10/5/1% level. for experience in previous 6 years: “*other exp.*”: employment in other occupation during the last six years, *unemp.*: unemployed at some point during the last six years

Source: Own calculations based on data of the SIAB 7514, BBSR [6], Statistisches Bundesamt [62]

**Table 9:** Results from proportional hazards estimation of occupation duration by occupation Source: Own calculations based on data of the SIAB 7514, BBSR [6], Statistisches Bundesamt [62]

Variable		Piecewise constant ph model—heterogeneneity					
		GN		HN		HN Assists.	
		Hazard ratio	z value	Hazard ratio	z-value	Hazard ratio	z value
Individual-level characteristics							
Wage (ref. 1st quartile)							
2nd quartile		0.56***	− 15.87	0.72***	− 8.33	0.68***	− 9.11
3rd quartile		0.32***	− 21.13	0.46***	− 15.65	0.37***	− 13.24
4th quartile		0.26***	− 17.78	0.45***	− 12.37	0.70***	− 3.28
Age (ref. 18–30)							
31–40		0.79***	− 6.05	0.75***	− 7.19	0.66***	− 9.22
41–50		0.65***	− 10.22	0.61***	10.03	0.54***	− 11.80
German citizen		1.03	0.42	0.99	− 0.20	0.94	− 0.85
Sex and volume of work (ref. male, part-time)							
Male, part-time		0.68***	− 4.77	1.23**	2.17	0.90	− 1.45
Female, full-time		1.03	0.64	1.15***	2.58	1.03	0.48
Female, part-time		0.65***	− 7.94	0.74***	− 4.66	0.70***	− 5.93
Firm-level characteristics							
Institution (ref. hospital)							
Nursing homes		1.10	1.09	1.46***	6.87	0.95	− 0.84
Health sector, rest		1.07	0.60	1.16**	2.58	0.78***	− 3.21
Social sector, rest		1.04	0.51	1.38***	5.14	0.93	− 1.05
Firm size (ref. < 20 employees)							
20–199		0.85***	− 3.47	0.83***	− 3.56	0.77***	− 4.71
Over 199		0.83***	− 2.96	0.70***	− 5.16	0.63***	− 6.14
Regional characteristics							
Population density		1.01***	5.55	1.01***	5.75	1.00**	2.19
Unemployment rate		0.99	− 1.70	0.96***	− 4.74	1.01	0.79
Labor market biography							
Experience in previous 6 years (ref. 3–6 years in care)							
Vocational training		0.98	− 0.21	0.76***	− 3.61	1.07	0.58
No care experience and							
Other exp	Unemp						
No	No	1.19	1.59	0.99	− 0.07	1.15	1.00
Yes	No	1.17**	2.44	1.15**	1.99	1.14*	1.68
No	Yes	1.44***	3.60	1.16	1.13	1.23*	1.69
Yes	Yes	1.40***	5.28	1.17**	2.05	1.21***	2.48
Up to 3 years experience in care and							
Other exp	Unemp						
No	No	0.93	− 0.88	0.94	− 1.21	1.30***	2.98
Yes	No	1.16**	2.02	1.15**	2.11	1.31***	3.19
No	Yes	1.42***	4.63	1.34***	4.14	1.34***	2.94
Yes	Yes	1.27***	3.69	1.30***	3.89	1.30***	3.33
3 to 6 years experience in care and							
Other exp	Unemp						
Yes	No	1.17*	1.76	1.05	0.67	1.02	0.19
No	Yes	1.28***	2.91	1.44***	5.04	1.33***	2.49
Yes	Yes	1.40***	3.36	1.55***	4.26	1.21	1.53
Employment episodes (ref. 1)							
2		1.10**	2.05	1.10*	1.64	0.93	− 1.36
3		1.04	0.73	1.06	0.90	0.94	− 0.97
4		1.09	1.50	1.13*	1.77	1.08	1.13
5		1.23***	2.97	1.25***	2.71	1.16*	1.89
6		1.47***	4.64	1.70***	5.39	1.47***	4.18
7 and more		1.81***	7.93	1.98***	7.03	1.61***	5.83
Further controls							
Year		X		X		X	
Federal states		X		X		X	
Time pieces		X		X		X	
Constant		0.00***	− 35.55	0.00***	− 39.00	0.00***	− 33.42
Theta		0.20		0.15		0.13	
Subjects		7,415		8,543		4,999	

*/**/*** significant at the 10/5/1% level

Experience in previous 6 years: “*other exp.*”: employment in other occupation during the last six years, *unemp.*: unemployed at some point during the last six years

**Table 10:** Results of proportional hazards estimation of occupation duration accounting for sex and children Source: Own calculations based on data of the SIAB 7514, BBSR [6], Statistisches Bundesamt [62]

Variable		Piecewise constant ph model—heterogeneneity			
		Women and men (1)		Women: with and without children (2)	
		Hazard ratio	z-value	Hazard ratio	z-value
Individual-level characteristics					
Wage (ref. 1st quartile)					
2nd quartile		0.62***	− 20.93	0.65***	− 17.35
3rd quartile		0.36***	− 31.78	0.41***	− 25.85
4th quartile		0.37***	− 22.67	0.38***	− 21.19
Age (ref. 18–30)					
31–40		0.73***	− 13.29	0.77***	− 9.60
41–50		0.59***	− 18.97	0.55***	− 19.90
German citizen		0.97	− 0.87	1.01	0.13
Women		0.95**	− 2.06	–	–
Part-time		0.68***	− 17.65	–	–
Volume of work (ref. full-time, no child)					
Part-time, no child		–	–	0.79***	-8.51
Full-time, child (ren)		–	–	0.87	-3.19
Part-time, child (ren)		–	–	0.47***	-20.92
Occupation (ref. geriatric nurse)					
Health nurse		0.86*	− 1.64	0.93	− 0.71
Health nurse assist		1.52***	4.77	1.49***	3.89
Firm-level characteristics					
Institution (ref. hospital)					
Nursing homes		1.28***	7.06	1.27***	6.16
Health sector, rest		1.11**	2.22	1.10*	1.67
Social sector, rest		1.23***	5.61	1.22***	5.03
Firm size (ref. < 20 employees)					
20–199		0.81***	− 6.99	0.82***	− 6.08
Over 199		0.71***	− 8.72	0.73***	− 7.22
Regional characteristics					
Population density		1.01***	8.14	1.01***	6.88
Unemployment rate		0.98***	− 3.28	0.98***	− 3.19
Labor market biography					
Experience in previous 6 years(ref. 3–6 years in care)					
Vocational training		0.85***	− 3.27	0.66***	− 4.94
No care experience and					
Other exp	Unemp				
No	No	1.08	1.23	0.99	-0.14
Yes	No	1.16**	3.88	1.10**	2.12
No	Yes	1.27**	3.62	1.20**	2.49
Yes	Yes	1.30***	6.88	1.24***	4.99
Up to 3 years experience in care and					
Other exp	Unemp				
No	No	0.99	− 0.26	0.97	− 0.60
Yes	No	1.20***	4.25	1.12**	2.37
No	Yes	1.32***	6.15	1.27***	4.77
Yes	Yes	1.25***	5.65	1.19***	3.91
3 to 6 years experience in care and					
Other exp	Unemp				
Yes	No	1.10*	1.66	1.04***	0.63
No	Yes	1.33***	5.73	1.34***	5.50
Yes	Yes	1.38***	5.20	1.38***	4.80
Employment episodes (ref. 1)					
2		1.03	1.07	1.10***	2.74
3		0.99	− 0.27	1.09***	2.34
4		1.07*	1.78	1.17***	3.60
5		1.19***	3.77	1.31***	5.28
6		1.50***	7.62	1.77***	9.58
7 and more		1.66***	10.13	1.86***	11.03
Further controls					
Year		X		X	
Federal states		X		X	
Time pieces		X		X	
Constant		0.00***	− 57.15	0.00***	− 52.64
Theta		0.21		0.18	
Subjects		18,322		14,870	

*/**/*** significant at the 10/5/1% level. Experience in previous 6 years: “*other exp.*”: employment in other occupation during the last six years, *unemp.*: unemployed at some point during the last six years.

**Table 11:** Results from proportional hazards estimation of occupation duration, baseline hazard modelled as quadratic polynomial with dummies for specific time intervals Source: Own calculations based on data of the SIAB 7514, BBSR [6], Statistisches Bundesamt [62]

Variable		Single risk estimation	
		Hazard ratio	z-value
Individual-level characteristics			
Wage (ref. 1st quartile)			
2nd quartile		0.65***	− 19.78
3rd quartile		0.39***	− 30.25
4th quartile		0.41***	− 21.06
Age (ref. 18–30)			
31–40		0.75***	− 12.48
41–50		0.62***	− 18.02
German citizen		0.97	− 0.68
Sex and volume of work (ref. male, full-time)			
Male, part-time		0.89***	− 2.66
Female, full-time		1.05*	1.70
Female, part-time		0.69***	− 11.14
Occupation (ref. geriatric nurse)			
Health nurse		0.85*	− 1.81
Health nurse assist		1.50***	4.71
Firm-level characteristics			
Institution (ref. hospital)			
Nursing homes		1.26***	6.75
Health sector, rest		1.10**	2.19
Social sector, rest		1.21***	5.30
Firm size (ref. < 20 employees)			
20–199		0.82***	− 6.72
Over 199		0.73***	− 8.46
Regional characteristics			
Population density		1.01***	7.81
Unemployment rate		0.98***	− 3.27
Labor market biography			
Vocational training		0.86***	− 2.98
Other exp	Unemp		
No	No	1.11	1.64
Yes	No	1.16***	3.88
No	Yes	1.28***	3.92
Yes	Yes	1.27***	6.55
Other exp	Unemp		
No	No	1.02	0.63
Yes	No	1.20***	4.56
No	Yes	1.35***	6.79
Yes	Yes	1.26***	6.11
Other exp	Unemp		
Yes	No	1.08	1.46
No	Yes	1.33***	5.92
Yes	Yes	1.37***	5.23
2		1.05	1.58
3		1.01	0.24
4		1.09**	2.30
5		1.20***	4.15
6		1.50***	7.91
7 and more		1.67***	10.77
Further controls			
Year		X	
Federal states		X	
Specific time pieces			
169–196 Days		1.43***	9.09
337–365 Days		2.41***	24.67
666–730 Days		1.74***	13.08
Time elapsed		0.999***	− 15.05
Time elapsed^2^		1.00***	7.24
Constant		0.00***	− 59.04
Theta		0.11	
Subjects		18,325	

*/**/***   significant at the 10/5/1% level. Experience in previous 6 years: “*other exp.*”: employment in other occupation during the last six years, *unemp.*: unemployed at some point during the last six years

Results on the factor variables “169–196 Days”, “337–365 Days”, and “666–730 Days” give the hazard ratios for the respective time intervals after entry. “Time Elapsed” and “Time Elapsed^2^” refer to analysis time since entry and analysis time since entry squared

## Footnotes

1 With the exception of nurses’ *intention to leave*, I focus on economic literature on nurses’ labor supply. There also exists a body of literature from the point of view of nursing studies. Regarding the latter, Hayes et al. [43] and Hayes et al. [42] provide broad literature overviews on nurses’ turnover.

2 In an earlier study, Simon et al. [61] presented analyses based on the German sample of the Nurses’ Early Exit-Study of 2003.

3 A handful of studies concern nurses’ leaving behavior in Germany [41], [38], [9], [37], [63]. Those studies focus on analyzing the extent of nurses’ exiting or the number of nurses still working in their training occupation after several years, apply basic descriptive analysis, and focus on distinct areas like federal states (an exception is Simon et al. [60], who studied factors associated with the intention to exit nursing, see above). The evidence is not entirely clear, though.

4 The data were accessed onsite at the Research Data Centre of the Federal Employment Agency (BA) at the Institute for Employment Research and via remote data access at the Research Data Centre.

5 Marginal employment is a form of tax-free employment in Germany. Over the time span of this analysis the maximum monthly earnings from a marginal employment rose from 325 to 400 Euros IAB-Forum [45].

6 Antoni and Jahn [5], Giannelli et al. [34], and Giannelli et al. [35] take a similar approach. The decreasing trend in failure events becomes apparent in Fig. 1.

7 A threshold of 90 days is a common choice for gap-length or reentry limit in studies that concern the German labor market and employ data on a daily basis, see e.g. Giannelli et al. [34] and Boockmann and Steffes [12].

8 A brief overview of the relevant definitions is given in Table 3 in the appendix.

9 This evolution over time is preserved if the baseline hazard is approximated via a quadratic polynomial with dummies for the time intervals covering half a year, one year, and two years after nurses (re)entered the occupation. Respective results are given in Table 11 in the appendix.

10 Regarding Europe, see Eurofound [28].

11 As my data do not contain information on the exact number of hours worked and full- as well as part-time employees enter my estimation, the estimated wage effect may be confounded, in part, by different amounts of hours worked in a part-time contract. However, if I only consider full-time contracts (not reported), the results for the wage effect hardly differ.

12 This conclusion does not change if, instead of studying the share of leavers, I analyze the predicted yearly hazard ratios, i.e. the “year effects” on leaving propensity, for the two groups after estimating a piecewise constant mixed proportional hazards model containing the same explanatory variables as model (1) reported in Table 1. Figure 6 in the appendix depicts the respective predicted hazard ratios.

13 This conclusion does not change if, instead of studying the share of leavers, I analyze the predicted semiannual hazard ratios, i.e. the “half-year effects” on leaving propensity, for the three groups after estimating a piecewise constant mixed proportional hazards model containing the same explanatory variables as model (1) reported in Table 1. Figure 7 in the appendix depicts the respective predicted hazard ratios.
